# Investigation and Alteration of Organic Acid Synthesis Pathways in the Mammalian Gut Symbiont Bacteroides thetaiotaomicron

**DOI:** 10.1128/spectrum.02312-21

**Published:** 2022-02-23

**Authors:** Nathan T. Porter, Johan Larsbrink

**Affiliations:** a Division of Industrial Biotechnology, Department of Biology and Biological Engineering, Chalmers University of Technology, Gothenburg, Sweden; b Wallenberg Wood Science Center, Department of Biology and Biological Engineering, Chalmers University of Technology, Gothenburg, Sweden; University of Georgia

**Keywords:** *Bacteroides*, fermentation, genetics, metabolism, organic acid

## Abstract

Members of the gut-dwelling *Bacteroides* genus have remarkable abilities in degrading a diverse set of fiber polysaccharide structures, most of which are found in the mammalian diet. As part of their metabolism, they convert these fibers to organic acids that can in turn provide energy to their host. While many studies have identified and characterized the genes and corresponding proteins involved in polysaccharide degradation, relatively little is known about *Bacteroides* genes involved in downstream metabolic pathways. Bacteroides thetaiotaomicron is one of the most studied species from the genus and is representative of this group in producing multiple organic acids as part of its metabolism. We focused here on several organic acid synthesis pathways in B. thetaiotaomicron, including those involved in formate, lactate, propionate, and acetate production. We identified potential genes involved in each pathway and characterized these through gene deletions coupled to growth assays and organic acid quantification. In addition, we developed and employed a Golden Gate-compatible plasmid system to simplify alteration of native gene expression levels. Our work both validates and contradicts previous bioinformatic gene annotations, and we develop a model on which to base future efforts. A clearer understanding of *Bacteroides* metabolic pathways can inform and facilitate efforts to employ these bacteria for improved human health or other utilization strategies.

**IMPORTANCE** Both humans and animals host a large community of bacteria and other microorganisms in their gastrointestinal tracts. This community breaks down dietary fiber and produces organic acids that are used as an energy source by the body and can also help the host resist infection by various pathogens. While the Bacteroides genus is one of the most common in the gut microbiota, it is only distantly related to bacteria with well-characterized metabolic pathways and it is therefore unclear whether research insights on organic acid production in those species can also be directly applied to the Bacteroides. By investigating multiple genetic pathways for organic acid production in Bacteroides thetaiotaomicron, we provide a basis for deeper understanding of these pathways. The work further enables greater understanding of Bacteroides–host relationships, as well as inter-species relationships in the microbiota, which are of importance for both human and animal gut health.

## INTRODUCTION

The gut microbiome of mammals is a dense and diverse community of organisms, including various bacteria, fungi, archaea, and their associated viruses (1). This community provides crucial nutrients to their hosts, including vitamins (1) and calories through intestinal cell absorption of microbial metabolites such as the organic acids (OAs) acetate, propionate, and butyrate (2). The gut microbiome also mediates resistance to colonization by pathogenic microorganisms, in part through organic acid production (3, 4). Factors such as host diet can influence the composition and the behavior of the community, leading to changes in microbial metabolite production and thus altered effects on the human or animal host (5).

The Bacteroidetes phylum generally comprises a large proportion of the bacteria in the gut microbiome in mammals, including humans. The Bacteroidetes, especially the intestinally dominant and intensively studied Bacteroides genus, are well known for the ability to degrade various fiber polysaccharides found in the human diet (6). As a result of fiber degradation, they produce acetate, propionate, and succinate as major end products (7). Bacteroides-generated OA proportions can vary depending on nutrients available in their environment. For instance, heme (from hemin) and vitamin B_12_ are cofactors for metabolic enzymes that lead to production of succinate and propionate, respectively; reduced concentrations of these components alters OA production accordingly (8, 9).

One of the main model organisms for the *Bacteroides*, B. thetaiotaomicron (*Bt*), was the first of this group to have its genome sequenced (10), which has enabled a multitude of studies focusing on its extensive machinery for both the degradation and synthesis of polysaccharide structures (11–15). However, many of the downstream fermentative pathways involved in producing OAs were described decades ago, before the genomic revolution (16). Thus, few studies have correlated the described enzyme activities with gene identities, prohibiting many followup genetic and biochemical studies of these enzymes and phenotypes. Large-scale efforts in automated and manual annotation of microbial genomes have led to increased understanding of their diverse metabolic capabilities, but the characterized genes/enzymes that the annotations are based on typically originate in model organisms such as E. coli, which is only distantly related to the *Bacteroides* (17). Indeed, recent efforts have produced genetic parts specifically for the *Bacteroides* because those from other organisms (e.g., E. coli) are not functional in this genus (18–20). In addition, the needs of the obligately anaerobic, polysaccharide-degrading Bacteroides in their native gut habitat likely differ significantly from better-studied model organisms and concomitantly affect both the available gene repertoire and possibly even enzyme function of homologous genes. Thus, annotations based on non-Bacteroidetes genes may be incorrect (21).

Recent studies have begun to focus on particular genes involved in various aspects of anaerobic metabolism in Bacteroides species, including the anaerobic respiratory chain (22), as well as various oxygen-sensitive enzymes (23, 24). In the present work, we expand on these efforts by identifying and validating which genes are involved in the natural production of multiple OAs in *Bt*, through genetic modifications, growth studies, and determination of OA profiles. This investigation of key metabolic pathways in Bacteroides permits greater understanding of how species from this genus behave in their native environment, and how their growth and metabolism affects the health of their hosts.

## RESULTS

**FIG 1:**
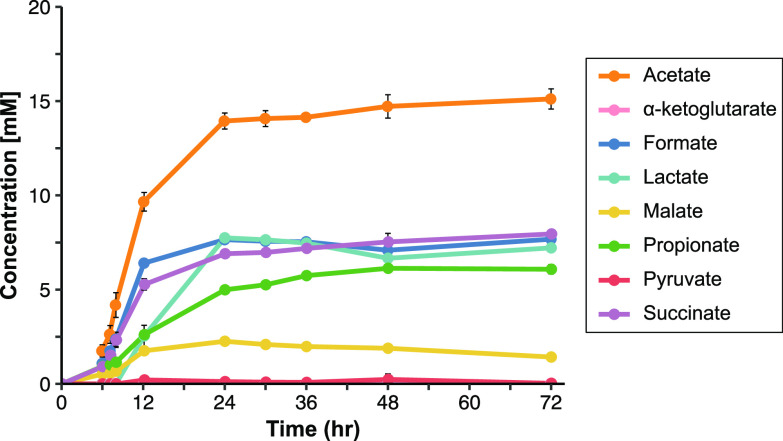
Wild-type (WT) *Bt* production of organic acids (OAs) in minimal medium with glucose over time. HPLC quantification of various OAs in supernatant from WT *Bt* (*n* = 3). Butyrate and fumarate were also assayed but not detected. Error bars show standard deviation.

Previous studies have reported production of a range of OAs by Bacteroides in various ratios, but with significant variation among strains and growth conditions (e.g., [24–26]). To obtain a reliable starting point, we first determined the OA production profile of *Bt* (VPI-5482 *tdk-* parent strain used for all subsequent work; wild-type, WT) using a standard minimal medium supplemented with glucose, from which samples taken for high-performance liquid chromatography (HPLC) analysis of OA production over a period of 3 days. Multiple OAs were readily detectable by 6 h postinoculation, with acetate being the most predominant OA by concentration (Fig. 1, Table S1 in the supplemental material). However, substantial quantities of other OAs were also produced at early time points, including formate, succinate, propionate, and malate. Only low levels of pyruvate and alpha-ketoglutarate were detected (less than 0.3 mM and 0.05 mM, respectively), and neither butyrate, fumarate, nor ethanol production were ever detected. We did not detect lactate until 12 h postinoculation (at which time optical density at 600 nm (OD600) of the culture had reached maximum levels), and lactate levels peaked by 24 h. This corresponded with a reduction in glucose, which by 12 h had dropped to ∼25% of starting levels and by 24 h was undetectable (Table S1). Later samples revealed only minor changes in OA concentrations.

**FIG 2:**
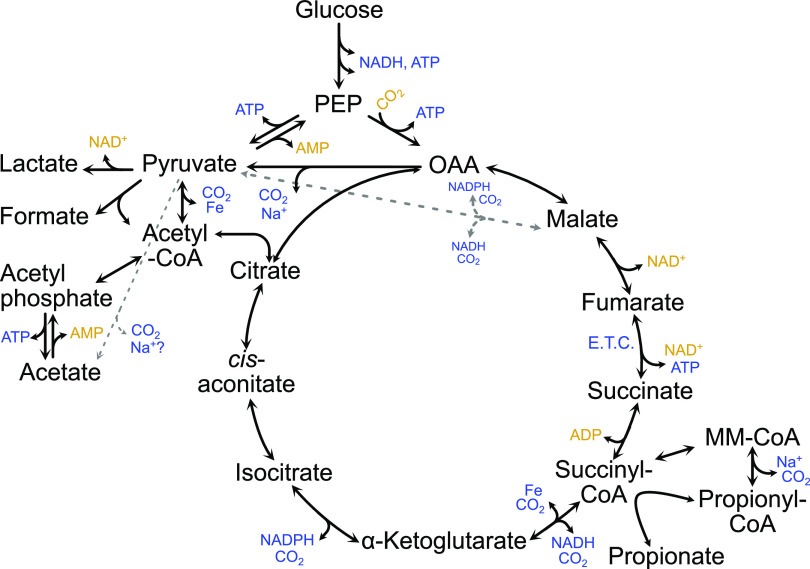
Model of *Bt* VPI-5482 OA production. Production of metabolites such as ATP is shown with blue font, whereas consumption of the same compounds is shown in gold. Glycolysis is inferred between glucose and PEP. PEP: Phospho-*enol*-pyruvate. OAA: Oxaloacetate. CoA: Coenzyme A. E.T.C.: Electron transport chain. MM-CoA: Methylmalonyl-CoA. Fe: Ferredoxin/flavodoxin.

As *Bt* produced multiple OAs, this provided an opportunity to confirm the genes involved in their production through a series of deletion mutants. We first created a working model of OA synthesis pathways (Fig. 2). We based our model on suggested pathways found previously in the literature for Bacteroides (16) and searched for relevant annotated genes and pathways in the *Bt* type strain in multiple online databases—Biocyc (27), IMG (28), Uniprot (29), and KEGG (30). While not all potential OA pathways are included in our model (e.g., branched chain fatty acids such as isovalerate detected in some studies) (31), the model encompasses central metabolism and includes all OAs detectable in our hands. A list of genes putatively involved in the identified metabolic pathways is included as Table S2 in the supplemental material. Some of these genes have been shown to be essential in large transposon screens (32, 33) (e.g., BT3692-3, putatively involved in acetate production). We chose to focus on deletion of a selection of genes annotated as involved in formate, lactate, propionate, or acetate production (shown in Fig. S1). As concentrations remained relatively stable at later time points in our initial WT strain time course experiment, samples from the generated mutant strains were collected at 48 h.

### Genes involved in formate production.

**FIG 3:**
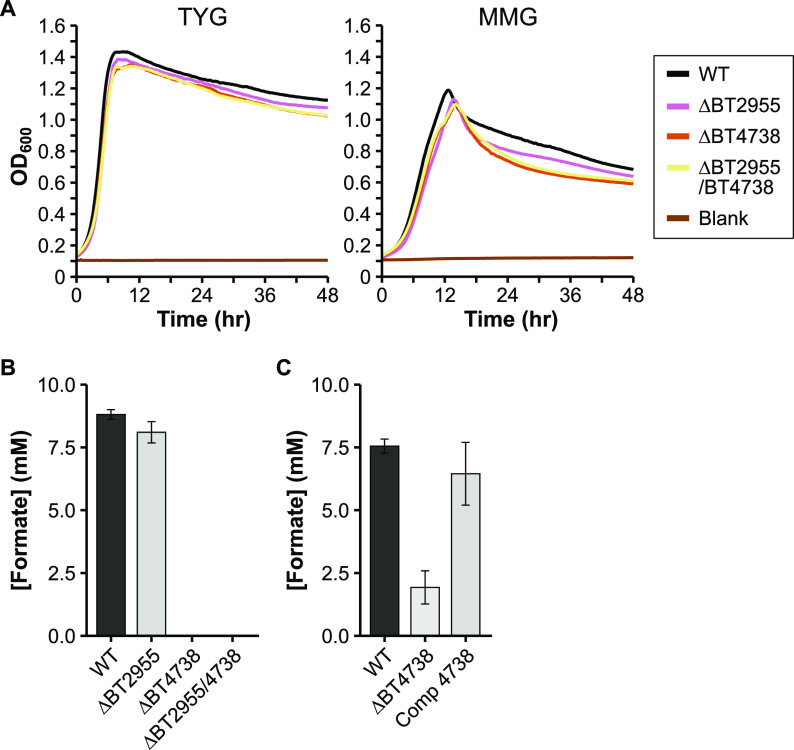
Characterization of potential formate production deletion mutants. A) Representative growth curves of the wild-type (WT) and mutant strains in either rich medium (TYG, left panel), or defined minimal medium with glucose (MMG, right panel). OD_600_: optical density at 600 nm. Average growth rate data can be found in Table S3 in the supplemental material. B) HPLC quantification of formate in WT and in various single or double mutants grown in minimal medium with glucose. *N* = 3 (except *n* = 2, ΔBT2955/BT4738). C) HPLC quantification of formate in WT, the ΔBT4738 strain, and the ΔBT4738 strain complemented with BT4738 (“Comp 4738”). N = 3. Error bars in panels B and C show standard deviation.

*Bt* carries two genes predicted to encode a pyruvate formate-lyase (PFL; also called formate acetyltransferase): BT2955 and BT4738. Each is located adjacent to a pyruvate formate-lyase activating enzyme (BT2956 and BT4737, respectively), which in E. coli is involved in post-translational regulation of PFL activity. The activated form of PFL carries out the CoA-mediated conversion of pyruvate to formate and acetyl-CoA. As we have identified no other genes annotated to carry out formate production, deletion of one or both of BT2955/BT4738 should eliminate formate *in vitro*. We grew each strain in a minimal medium with glucose as the sole carbon source and analyzed OA content in culture supernatant as mentioned earlier. Whereas the ΔBT2955 strain revealed no substantial difference in formate production from the WT strain (Fig. 3B), the ΔBT4738 and ΔBT2955/ΔBT4738 strains produced no formate, supporting the role of BT4738 as a PFL. This annotation was also recently confirmed by others (34). Interestingly, deletion of BT4738 also resulted in reduced lactate formation, as well as increased production of propionate and succinate (Table 1). Deletion of BT4738 resulted in only a slight growth defect when grown in minimal medium with glucose, perhaps because redox balance and ATP production are not directly affected (Fig. 3A, Table S3). To further confirm the PFL role of BT4738, we restored formate production by providing BT4738 in *trans* (Table S4, Fig. 3C). Interestingly, the ΔBT4738 strain exhibited lower but not abolished formate production in this repeat experiment. The ΔBT4738 strain did not produce formate in additional experiments in our hands (data not shown), nor did this occur in work by others (34). We speculate that BT2955, which is normally off *in vitro*, may somewhat compensate for the lack of the normally abundantly expressed BT4738 (35) (see also WT expression levels in Table S2).

**TABLE 1:** Organic acid concentrations (mM ± standard deviation) at 48 h for the wild-type (WT) and various mutant strains

Strain	Acetate	α-keto glutarate	Formate	Lactate	Malate	Propionate	Pyruvate	Succinate
WT	15.78 ± 0.52	0.02 ± 0.00	8.81 ± 0.19	9.37 ± 0.42	2.03 ± 0.12	4.69 ± 0.34	0.10 ± 0.00	8.66 ± 0.15
ΔBT2955	15.42 ± 0.37	0.02 ± 0.01	8.10 ± 0.43	9.74 ± 0.55	2.28 ± 0.13	4.45 ± 0.02	0.13 ± 0.02	8.46 ± 0.12
ΔBT4738	14.87 ± 0.40	0.01 ± 0.00	ND^a^**	6.90 ± 0.72	2.27 ± 0.16	5.20 ± 0.32	0.22 ± 0.03	11.07 ± 0.16**
ΔBT2955 /4738^*b*^	15.61 ± 1.17	0.01 ± 0.00	ND**	6.63 ± 0.33*	2.37 ± 0.06	5.47 ± 0.83	0.19 ± 0.00**	11.25 ± 0.73
ΔBT1575	16.19 ± 0.64	0.02 ± 0.02	9.14 ± 0.69	7.69 ± 0.73	1.88 ± 0.33	5.21 ± 0.65	0.14 ± 0.07	8.73 ± 0.70
ΔBT4455-7	15.99 ± 0.34	0.02 ± 0.00	8.62 ± 0.81	9.76 ± 0.19	2.26 ± 0.11	4.64 ± 0.15	0.14 ± 0.03	8.82 ± 0.53
ΔBT1575 /4455-7	16.59 ± 0.74	0.02 ± 0.02	9.60 ± 0.91	8.28 ± 0.45	1.86 ± 0.44	4.97 ± 0.60	0.17 ± 0.05	8.90 ± 0.32
ΔBT1450	16.48 ± 0.70	0.01 ± 0.00	9.17 ± 0.39	8.27 ± 0.72	1.78 ± 0.17	5.23 ± 0.60	0.12 ± 0.02	8.03 ± 0.33
ΔBT1686-9	16.25 ± 0.39	0.02 ± 0.01	7.92 ± 0.14*	9.31 ± 0.62	2.08 ± 0.31	ND*	0.12 ± 0.02	15.00 ± 0.27**
ΔBT1917	16.07 ± 0.02	0.02 ± 0.01	9.09 ± 0.34	9.24 ± 0.65	2.07 ± 0.22	5.01 ± 0.22	0.14 ± 0.00**	8.10 ± 0.31
ΔBT2090-1	15.77 ± 0.29	0.06 ± 0.01	8.75 ± 0.49	8.98 ± 1.08	1.88 ± 0.76	ND*	0.17 ± 0.02	14.77 ± 0.60*
ΔBT3193	1.72 ± 0.25**	ND	ND**	2.73 ± 0.40**	ND*	ND*	1.17 ± 0.35	0.74 ± 0.35**
ΔBT1969	14.61 ± 0.94	0.03 ± 0.01	7.23 ± 0.61	8.75 ± 1.10	2.37 ± 0.31	5.32 ± 1.01	0.14 ± 0.02	10.40 ± 0.49
ΔBT1820	17.04 ± 0.61	0.01 ± 0.01	8.30 ± 1.29	8.44 ± 0.46	2.01 ± 0.27	5.27 ± 0.39	0.15 ± 0.01**	8.42 ± 0.91
ΔBT1822	16.04 ± 1.14	0.01 ± 0.00	9.47 ± 0.49	8.63 ± 0.56	1.75 ± 0.13	5.16 ± 0.54	0.15 ± 0.04	8.08 ± 0.60
ΔBT1820 /1822	16.00 ± 0.35	0.02 ± 0.01	8.93 ± 0.67	9.33 ± 0.26	2.04 ± 0.21	4.72 ± 0.19	0.11 ± 0.00	9.09 ± 0.59

a ND: Not detected; butyrate and fumarate were not detected in any samples.*, *P* < 0.05; **, *P* < 0.01 according to Welch two-sample t test adjusted for multiple comparisons with the Benjamini/Hochberg FDR method.

b n = 2; all others *n* = 3.

### Genes involved in lactate production.

**FIG 4:**
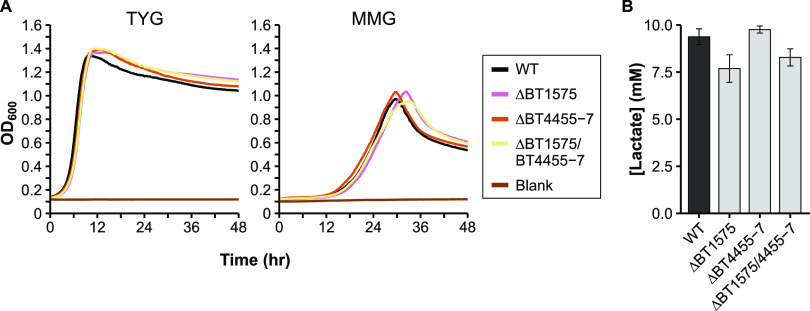
Characterization of potential lactate production deletion mutants. A) Representative growth curves of the wild-type (WT) and mutant strains in either rich medium (TYG, left panel), or defined minimal medium with glucose (MMG, right panel). OD_600_: optical density at 600 nm. Average growth rate data can be found in Table S3 in the supplemental material. B) HPLC quantification of lactate in culture supernatant from the WT and in various single or double mutants grown in minimal medium with glucose. N = 3. Error bars in panel B show standard deviation.

d-lactate dehydrogenase converts pyruvate to d-lactate while regenerating NAD^+^. *Bt* encodes one annotated lactate dehydrogenase (BT1575) with 50% amino acid identity to the E. coli *ldhA* gene. However, deletion of BT1575 only decreased lactate production to ∼75% of WT levels (Fig. 4B), prompting us to search for another metabolic route to lactate. BT4455-7 form a predicted complex which is homologous to a 3-gene lactate dehydrogenase complex examined in both Shewanella oneidensis and E. coli (36, 37). While this enzyme complex favors consumption rather than production of lactate in those organisms, both BT1575 and BT4455-7 increase in expression in the transition from exponential to stationary phase (Table S2 in the supplemental material 35), mirroring the rise in lactate in our WT time course (Fig. 1). We deleted BT4455-7 individually and in combination with BT1575. However, the double mutant did not exhibit further reductions in lactate compared to the ΔBT1575 strain (Fig. 4B). Deletion of either or both putative lactate dehydrogenases also had no effect on growth rates, indicating that *Bt* can easily compensate for the slight reduction in lactate (Fig. 4A), and likely a separate lactate-generating enzyme that escapes current annotations is present in the genome.

### Genes involved in propionate production.

Propionate production from succinate likely proceeds in several steps (*Bt* locus tags for gene candidates in parentheses): (i) succinyl-CoA synthetase (BT0787-8) converts succinate to succinyl-CoA in the tricarboxylic acid (TCA) cycle, (ii) methylmalonyl-CoA mutase (BT2090-1) converts succinyl-CoA to the branched methylmalonyl-CoA, (iii) methylmalonyl-CoA decarboxylase (BT1686-9) decarboxylates to propionyl-CoA, and (iv) succinate CoA transferase (BT3193) transfers the CoA from propionyl-CoA to succinate to regenerate succinyl-CoA and produce propionate. *Bt* also encodes a gene annotated as a methylmalonyl-CoA epimerase (BT1685), which would convert between *R*- and *S*- forms of methylmalonyl-CoA and may also be necessary before decarboxylation. Two other genes, BT1450 and BT1917, are annotated as propionyl-CoA carboxylase beta chains (subunit of methylmalonyl-CoA decarboxylase, similar to BT1686), and are found within loci predicted to encode biotin-dependent carboxylases, similar to BT1686-9. We generated deletion mutants for BT1450, BT1917, BT2090-1, and BT3193, and measured OA production as before. A deletion mutant for BT1686-9 has already been generated and tested (38). No propionate was detected in gnotobiotic mice monocolonized with the ΔBT1686-9 strain, indicating a bacterial deficiency in propionate production. However, we chose to measure OA production of this strain *in vitro* to eliminate any contribution of host uptake of propionate and to quantify other OAs produced.

**FIG 5:**
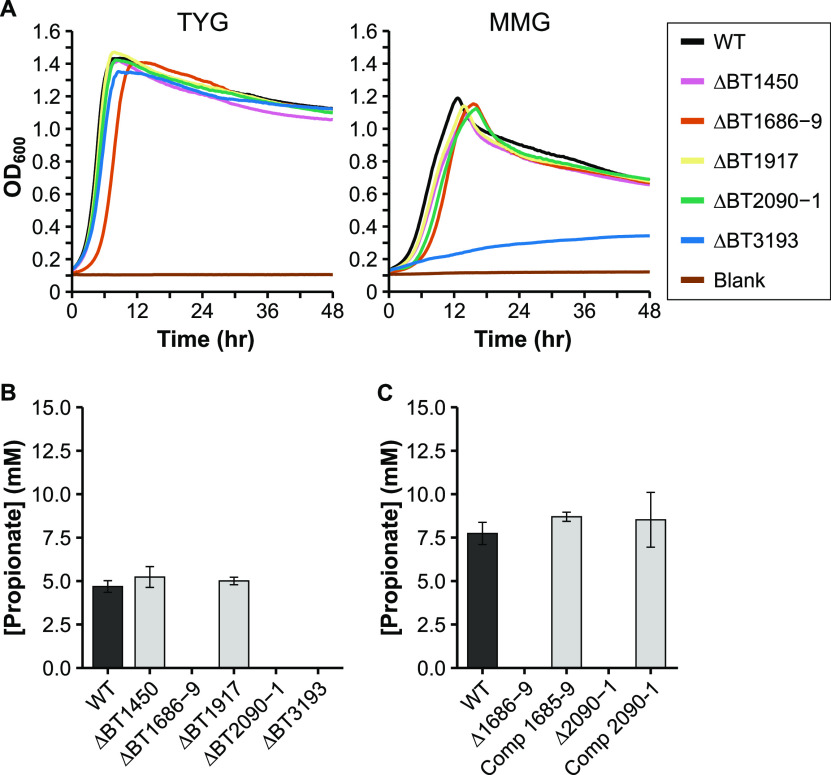
Characterization of potential propionate production deletion mutants. A) Representative growth curves of the wild-type (WT) and mutant strains in either rich medium (TYG, left panel), or defined minimal medium with glucose (MMG, right panel). OD_600_: optical density at 600 nm. Average growth rate data can be found in Table S3 in the supplemental material. B) HPLC quantification of propionate in culture supernatant from the WT strain and in various single or double mutants grown in minimal medium with glucose. N = 3. C) HPLC quantification of propionate in the WT, the ΔBT1686-9 strain, the ΔBT2090-1 strain, and their complemented strains (“Comp 1685-9” and “Comp 2090-1,” respectively) grown in minimal medium with glucose. N = 3. Error bars in panels B and C show standard deviation.

The ΔBT1450 and ΔBT1917 strains exhibited OA profiles and growth rates similar to the WT strain, indicating they are not necessary for propionate production in *Bt* (Fig. 5A and B). However, future deletion or biochemical characterization of the whole locus for each enzyme (BT1448-50, BT1915-17) may be needed to determine actual enzyme function. In contrast, the three other deletion strains produced no propionate. The ΔBT1686-9 and ΔBT2090-1 strains increased succinate output by roughly the same amount as the reduction in propionate (Table 1), while exhibiting no growth defect in minimal medium (only in rich medium), confirming their contribution to propionate synthesis by decarboxylation of succinate (Fig. 5A). Complementation of these two mutants restored elevated propionate levels (Fig. 5C, Table S4). On the other hand, the ΔBT3193 strain grew very poorly in minimal medium with glucose (Fig. 5A), and only reached OD_600_ 0.2–0.3 for our OA production analysis (other strains reaching ∼OD_600_ 1.0 at 48 h). This defect could be rescued by addition of rich media components (in the paired TYG medium). Still, the ΔBT3193 strain produced no propionate during its stunted growth. As it is annotated as a coenzyme A transferase, BT3193 likely fulfills its predicted role as a succinate CoA transferase.

### A gene connecting the reductive and oxidative branches of metabolism.

**FIG 6:**
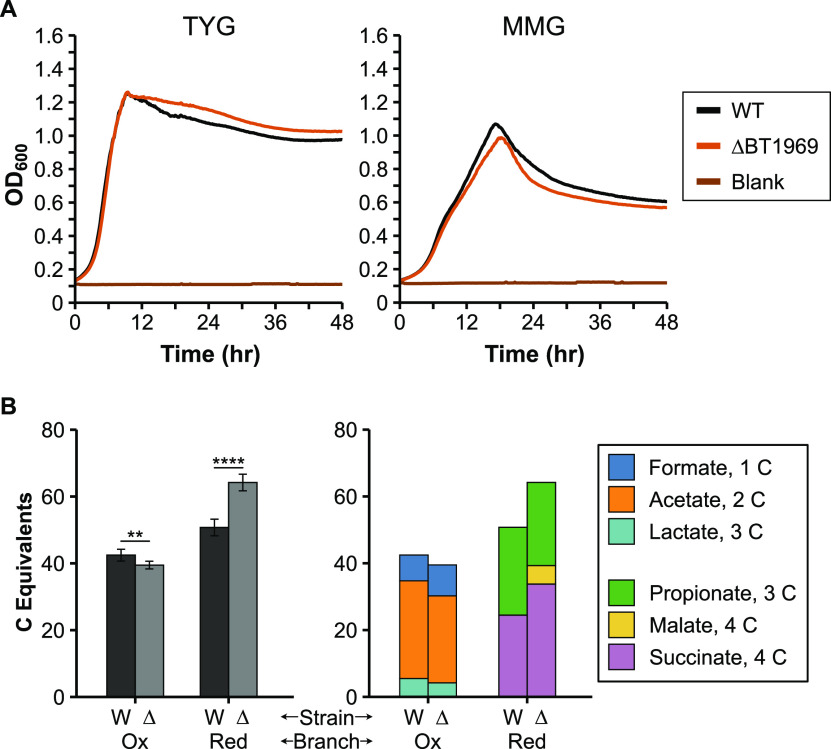
Characterization of growth and oxidative versus reductive branch metabolite production in the wild-type (WT) and ΔBT1969 strains. A) Representative growth curves of WT and ΔBT1969 strain in either rich medium (TYG, left panel), or defined minimal medium with glucose (MMG, right panel). OD_600_: optical density at 600 nm. Average growth rate data can be found in Table S3 in the supplemental material. B) Oxidative branch metabolites (pyruvate, formate, lactate, and acetate) and reductive branch metabolites (malate, succinate, and propionate) were quantified by HPLC with culture supernatants from the two strains grown in minimal medium with glucose (*n* = 6 each). Concentrations of each OA were multiplied by the number of carbon (C) atoms per molecule to calculate “C equivalents.” Oxidative and reductive branch C equivalents were separately pooled for comparison. The left panel shows metabolites pooled by branch. Error bars show standard deviation. Samples were compared using the Welch two-sample *t* test. **, *P* < 0.01. ****, *P* < 0.0001. The right panel shows C equivalents for individual OAs. W: wild type; Δ: ΔBT1969; Ox: oxidative branch; Red: reductive branch. Individual OA concentrations and standard deviations are shown in Table S5.

We also tested a “mid-pathway” gene predicted to connect the oxidative and reductive branches of the TCA cycle and result in production of pyruvate. Because there are multiple pathways for producing and consuming this central metabolite, we speculated that more general changes in OA production would be evident after deletion of this gene. BT1969 is an annotated NADP^+^-dependent malate dehydrogenase with 50% amino acid identity to *maeB* (malic enzyme) from E. coli. MaeB decarboxylates malate to produce pyruvate, CO_2_, and NADPH, shunting carbon from reductive branch end products to oxidative branch metabolites. Deletion of BT1969 should thus sever this connection between the reductive and oxidative branches and favor an OA profile with a greater proportion of reductive branch metabolites than oxidative branch metabolites. In our initial assay, the ΔBT1969 strain grew comparably to the WT strain (Fig. 6A) and produced equally low levels of pyruvate. However, levels of other organic acids from the oxidative branch of central metabolism (lactate/formate/acetate) decreased, whereas levels of organic acids from the reductive branch (malate/succinate/propionate) increased in abundance (Table 1). To compare carbon flow to the oxidative and reductive branches between the WT and ΔBT1969 strain, we normalized each OA concentration to the number of carbon (C) atoms per molecule (“carbon equivalents” (C equivalents), e.g., acetate corresponds to 2 C equivalents). Pooling these normalized values by reductive or oxidative branch revealed a shift toward reductive branch OAs in the ΔBT1969 strain. To confirm this metabolic shift toward the reductive branch, we repeated this experiment with a larger sample size (*n* = 6). Metabolite profiles in this repeat experiment generally mirrored those of the original experiment, though with some individual organic acid concentrations changing (e.g., lactate concentration was higher in the ΔBT1969 cultures than in the wild-type cultures in this case) (Table S5 in the supplemental material). This continues to fit a model wherein OA concentrations may vary within the same metabolic branch, as deletion of BT1969 should only remove a connection between reductive and oxidative branches. Normalizing OA concentrations by size and metabolic branch confirmed a statistically significant shift of metabolism toward the reductive branch in the ΔBT1969 strain (Fig. 6B), supporting the role of BT1969 as a malic enzyme.

### Genes involved in acetate production.

**FIG 7:**
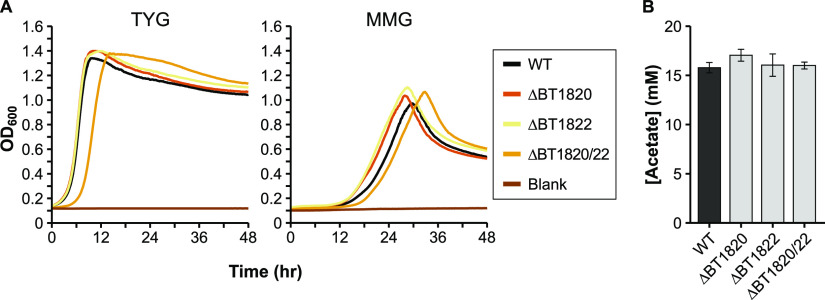
Characterization of potential acetate production deletion mutants. A) Representative growth curves of the wild-type (WT) and mutant strains in either rich medium (TYG, left panel), or defined minimal medium with glucose (MMG, right panel). OD_600_: optical density at 600 nm. Average growth rate data can be found in Table S3 in the supplemental material. B) HPLC quantification of acetate in culture supernatant from the WT strain and in various single or double mutants (*n* = 3 each) grown in minimal medium with glucose. Error bars in panel B show standard deviation.

Pyruvate dehydrogenase (quinone; PDHq) converts pyruvate to acetate and CO_2_ while donating electrons to quinone. *Bt* encodes a set of 2 short genes (BT1820, BT1822) that each contain motifs found in an intact PDHq in E. coli and other organisms. These two genes are separated by a transposase (BT1821), all 3 of which are upregulated in stationary phase (Table S2 in the supplemental material) (35). As the Bacteroides are replete with invertible and phase variable elements (15, 39) we initially erroneously assumed that BT1821 provided some regulatory function for BT1820 and BT1822, which together should encode a complete enzyme. However, deletion of one or both open reading frames (BT1820/BT1822) led to no changes in growth rates (Fig. 7A) or to OA production (Fig. 7B, Table 1). A subsequent comparison to PDHq-annotated genes in *Bt* genomes in the IMG database (28) revealed single, intact genes in other strains. For instance, in the B. thetaiotaomicron 7330 strain, the gene for PDHq is 100% identical to a fused BT1820/BT1822 at the nucleotide level when removing BT1821 and a short flanking sequence. An analysis of the VPI-5482 genome uncovered an additional two copies of the BT1821 transposase with ≥99% identity to BT1821, though they did not appear to be inserted into a coding sequence. Clearly the putative PDHq is not essential to *Bt*, but it is yet unclear whether its function in other strains is as annotated.

With a disrupted PDHq gene, *Bt* VPI-5482 likely produces most or all of its acetate from acetyl-CoA using the essential BT3692-3 locus. BT3692 is annotated as a phosphate acetyltransferase (acetyl-CoA → acetyl phosphate) whereas BT3693 is a putative acetate kinase (acetyl phosphate → acetate), producing ATP and the secreted product acetate. Both genes are expressed at moderately high levels during early and late exponential growth (Table S2). While we could not delete these essential genes (32, 33), we attempted to confirm their role in acetate production by downmodulating their expression.

We selected five native promoters with consistently low expression (see Materials and Methods), as well as a set of four synthetic promoters of various expression levels (19) to replace the native P_BT3692_ promoter. Initial attempts to assemble the necessary vectors in E. coli failed, possibly due to promoter-driven expression of gene fragments from the flanking regions (data not shown). To facilitate our work, we generated a new Golden Gate-compatible suicide plasmid system based on pExchange-tdk (40)—the plasmid used for gene deletions in this study. This new system consists of a plasmid for cloning new parts (pNTP201), as well as two plasmids each carrying part of the original pExchange-tdk plasmid (pNTP202, pNTP203). The pExchange-tdk plasmid was divided in the middle of the ampicillin resistance gene to allow selection on ampicillin for the assembled plasmid, similar to another *Bacteroides* plasmid system using a different integration mechanism (19). Plasmids were engineered to remove internal BsaI and BsmBI restriction sites, and both the plasmid parts and the final assembled plasmid contain terminators flanking each side of the insert site to reduce potential effects of the promoters in E. coli. Fully assembled plasmids are designated as derivatives of pExchange2 (Fig. S2 in the supplemental material).

**FIG 8:**
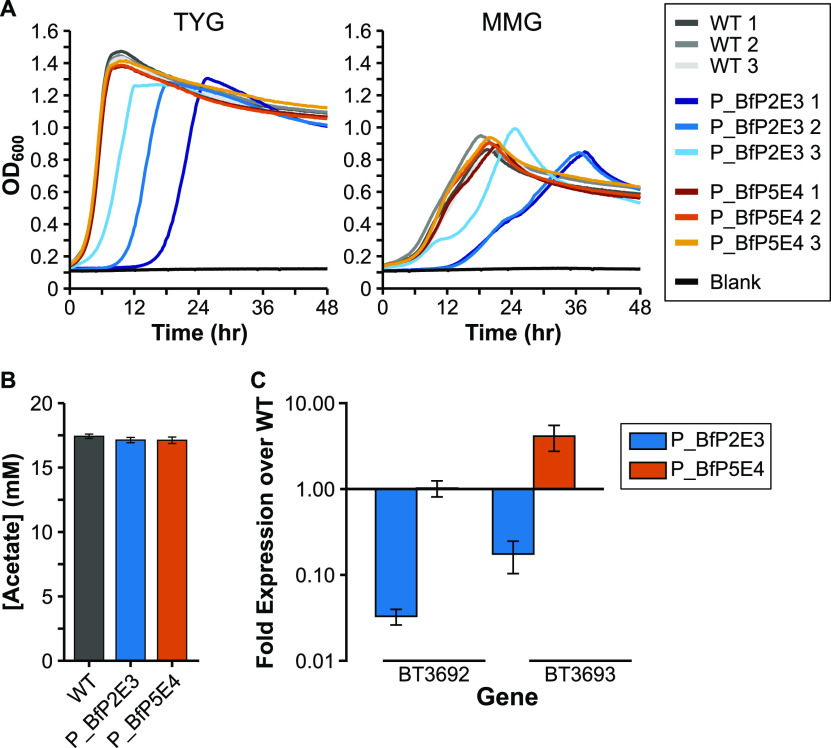
Characterization of mutants with P_BT3692_ promoter replacement. A) Growth curves of independent clones (*n* = 3 each) of the wild-type (WT) and each BT3692 promoter mutant strain in either rich medium (TYG, left panel), or defined minimal medium with glucose (MMG, right panel). OD_600_: optical density at 600 nm. Average growth rate data can be found in Table S3 in the supplemental material. B) HPLC quantification of acetate in culture supernatant from the WT and the BT3692 promoter mutants (*n* = 3 each) grown in minimal medium with glucose. C) BT3692/BT3693 gene expression over WT levels in the promoter mutant strains grown in panel B. Error bars in panels B and C show standard deviation.

Each promoter was successfully incorporated with the P_BT3692_ flanking regions into our novel pExchange2 vector. While all vectors could successfully integrate at the P_BT3692_ target site in the *Bt* genome, only the control vectors encoding promoters for higher gene expression (P_BfP2E3 and P_BfP5E4) were successful at replacing the native promoter upon plasmid removal (data not shown). We then attempted to correlate BT3692-3 gene expression in the WT strain and these two promoter replacement strains with levels of acetate production *in vitro*. As production of acetate provides ATP as well as critical cell metabolites (e.g., acetyl-CoA), a lower rate of acetate production should slow cell growth. The same individual clones (*n* = 3) of each strain were used for growth rate analysis as well as for measuring BT3692-3 gene expression and OA concentrations in the culture supernatant. Growth assays showed that the strain with the P_BfP2E3 promoter displayed a growth defect in both rich and minimal medium compared to the WT and P_BfP5E4-containing strains (Fig. 8A). In addition, analysis of gene expression in the P_BfP2E3 promoter strain revealed an ∼30-fold and ∼7-fold reduction in BT3692 and BT3693 gene expression, respectively, compared to the WT strain (Fig. 8C). These data corroborate our hypothesis of acetate-mediated growth rate. However, whereas the P_BfP2E3 promoter strain variably produced more formate and lactate, acetate concentrations from the cell supernatants were very similar after 48 h of growth (Fig. 8B, Table S6 in the supplemental material). Presumably, the P_BfP2E3 promoter strain produces a similar mix of OAs to the WT as it grows but takes longer to reach its maximum levels because of limited available cellular ATP. Samples taken at ∼OD_600_ 0.6 (the time of gene expression) had barely detectable acetate concentrations (data not shown), but later intermediate time points in future experiments could address whether there is a difference in OA production during early growth stages.

## DISCUSSION

In this study, we investigate central metabolic pathways of the type strain of B. thetaiotaomicron, a model organism for studying the gut-dwelling *Bacteroides* genus. The phylum Bacteroidetes is itself only distantly related to the Proteobacteria (17) such as the intensively-studied model E. coli, and thus even proteins with significant homology may have divergent functions. For instance, in another Proteobacterial species, Shewanella oneidensis, a gene annotated as a fumarate reductase was characterized and found to be a urocanate reductase (41). Misannotations due to overprediction (assignment to more specific enzyme functions than can be supported) is very common in genome annotations based on sometimes low similarities (21, 42). In our work, we have identified three genes annotated as encoding the beta chain of propionyl-CoA carboxylase. However, deletion of only one of them, BT1686 (as part of a larger deletion), leads to abolishment of propionate production (Fig. 5). Rather than specifically propionyl-CoA carboxylases, the two other loci, BT1448-50 and BT1915-17, likely encode other biotin-dependent carboxylases.

Furthermore, deletion of two potential lactate dehydrogenases was not sufficient to abolish lactate production in our assays (Fig. 4), and other lactate dehydrogenase or lactate-generating enzymes are likely present in the genome. One potential source of these could be the two genes annotated as malate dehydrogenases: BT2510 and BT3911. Malate and lactate dehydrogenases are part of the same enzyme superfamily and share many features that complicate correct annotation of these genes (43). Future genetic and biochemical work is needed to characterize these and other genes/loci to determine their specific functions, especially in key microbes distantly related to the best-studied model organisms.

Although our efforts to reduce acetate production through downmodulated BT3692-3 expression were not as successful as anticipated, we generated a new Golden Gate-compatible plasmid system as part of this aim. Our “pExchange2” system allowed us to transform an insert into E. coli that would otherwise fail, perhaps due to added terminators to reduce transcription of the genes flanking our promoter of interest. Additionally, using Golden Gate cloning and carefully choosing the overhangs for the 3 inserts let us avoid splicing by overlap extension (SOE)—a common technique for assembling flanking regions for deletion vectors. The pExchange2 system is compatible with a recently developed set of *Bacteroides* promoters and ribosome binding site sequences (19), as we used a subset of these in our study (Fig. 8). We hope this new tool will be of benefit to the research community.

B. thetaiotaomicron has become a major model organism of the gut microbiota. Its strategies to successfully compete for both dietary fiber and human-derived glycans is by now well-studied (44), but how it produces the end products from this process is poorly understood. Our collective data serves to clarify the picture of the central metabolism of *Bt*, with both verification and rejection of previous gene annotations. While we focused much of this work on deleting genes of interest, both follow-up genetic studies and biochemical work are necessary to fully validate the function of many of these enzymes and define their role in the bacterium’s response to its changing environmental conditions. A clearer understanding of the metabolism of this major group from the mammalian gut provides a way to take advantage of these pathways for industrial applications or to benefit human gut health.

## MATERIALS AND METHODS

### Media and growth conditions.

Strains and plasmids used in this study are listed in Table S7 in the supplemental material. Escherichia coli was routinely grown in LB medium at 37°C, with antibiotics as appropriate: ampicillin 100 μg/mL, chloramphenicol 25 μg/mL. Bacteroides thetaiotaomicron was routinely grown in TYG medium (10 g/L tryptone, 5 g/L yeast extract, 4 g/L glucose, 100 mM potassium phosphate [pH 7.2], 15 mM NaCl, 8.5 mM [NH_4_]_2_SO_4_, 4 mM l-cysteine, 200 μM l-histidine, 100 μM MgCl_2_, 72.1 μM CaCl_2_, 1.9 μM hematin, 1.4 μM FeSO_4_ × 7H_2_O, 1 μg/mL vitamin K_3_, and 5 ng/mL vitamin B_12_), or a paired minimal medium with additional glucose (increased to 5 g/L) and lacking tryptone and yeast extract. For solid media, strains were grown on TYG agar plates (TYG medium containing 15 g/L agar), or blood agar plates (BHI agar plates containing 10% defibrillated horse blood; Håtunalab AB, Bro, Sweden), as indicated for each experiment. Antibiotics in Bacteroides media were included as appropriate: gentamicin (200 μg/mL), erythromycin (25 μg/mL), tetracycline (2 μg/mL), and/or floxuridine (200 μg/mL). For microplate-based growth assays, cells were cultured anaerobically at 37°C in a vinyl anaerobic chamber (Coy Laboratory Products, Grass Lake, MI, USA) using a gas mix of 10-10-80% H_2_-CO_2_-N_2_. For other experiments, a Don Whitley A95 anaerobic cabinet (Don Whitley Scientific Ltd, Bingley, West Yorkshire, UK) was used with the same gas mix. B. thetaiotaomicron was grown anaerobically at 37°C unless otherwise noted below.

### Identification of native B. thetaiotaomicron promoters with stable, low expression.

To identify native promoters that were stably expressed in a variety of *in vitro* and *in vivo* conditions, data from several transcriptomic studies were analyzed (45–48). Data sets from each study were retrieved from the NCBI Gene Expression Omnibus repository. Gene expression within each sample was normalized by dividing each value by the total gene expression value for the sample and then multiplied by 1E5. Genes with a low average expression level (0–10 units) across the several data sets were then screened for first-in-operon genes identified by Westover and colleagues (49). The promoter for BT4618 was also added to this list, as it was previously used for low-level expression (20). Resulting genes were binned into approximately 0–1 units, 2–5 units, and 6–10 units. The NormFinder algorithm (50) was used to identify genes in each bin that were most consistently expressed, and the promoter regions for several of these genes were visually inspected before selection for use in this study. In addition, four synthetic promoters with a range of expression levels, coupled with a synthetic ribosome binding site, were included (19). All promoter sequences used are included in Table S8 in the supplemental material.

### Golden Gate cloning.

Parts for various plasmids were amplified by PCR, ordered as sets of oligonucleotides, or directly synthesized. Before use, paired oligonucleotides were annealed in T4 DNA Ligase buffer with the following program: 98°C for 5 min; then cooling by 1°C every 30 s until reaching 12°C. pNTP201 was assembled with traditional enzymatic restriction and ligation of PCR products. Parts for subsequent plasmids were incorporated into pNTP201 by Golden Gate cloning using FastDigest Esp3I (isoschizomer of BsmBI) and the following cycling protocol: 37°C for 4 min; 25 cycles of: 37°C for 1 min, 16°C for 2 min; 50°C for 10 min; and 80°C for 10 min. Final plasmids for conjugation to B. thetaiotaomicron were assembled by Golden Gate cloning using various plasmid parts, PCR products, and/or annealed oligonucleotides. For these, a longer cycling protocol was used: 37°C for 4 min; 40 cycles of: 37°C for 1 min and 16°C for 2 min; 50°C for 10 min; and 80°C for 10 min. 500 bp of sequence flanking each side of the BT3692-3 promoter was assembled with one of various promoter regions into final pExchange2-based vectors.

### Genetic manipulation of B. thetaiotaomicron.

Gene deletions and promoter swapping using the B. thetaiotaomicron VPI-5482 *tdk*- parental strain were carried out as described previously (40). For deletion constructs, 500-750 bp flanking sequences from each side of a target gene or locus were assembled and ligated into pExchange-tdk by traditional restriction-ligation cloning. Flanking sequences were designed to leave open reading frames intact while deleting most or all of the coding sequence. Primers are listed in Table S8 in the supplemental material. Constructs for gene/locus deletion or promoter replacement were transformed into E. coli S17-1 λ*pir* by electroporation and selected on LB agar with ampicillin. For conjugation to B. thetaiotaomicron, mid-log cultures of plasmid-containing E. coli strains and B. thetaiotaomicron *tdk-* or derivative strains were washed and plated together on blood agar plates without antibiotics for 1 day of aerobic growth at 37°C. The cell mass was then scraped up and diluted, and cells were twice plated on blood agar plates with gentamicin and erythromycin and grown anaerobically at 37°C for 2–3 days. Up to 10 merodiploid colonies were grown in TYG medium without antibiotics before counterselection by twice plating on blood agar plates containing floxuridine. Resulting clones were picked into TYG medium and screened by PCR for the appropriate deletion. The ΔBT1686-9 strain from (38) exhibited a large and small colony morphology on TYG agar plates, but no difference in size on blood agar plates. Since both clones tested positive for deletion of BT1686-9 (data not shown), and since the smaller morphology clone did not grow well overnight, we chose to work with the large colony morphology.

### Identification of organic acids from cell supernatants.

Bacterial strains were streaked on TYG agar and grown at 37°C for 2–3 days. Three or more isolated colonies were then picked into TYG liquid medium and grown overnight. As various strains exhibited some amount of growth defect, optical density at 600 nm (OD_600_) was measured for each overnight culture. A culture amount that approximately corresponded to 50 μL of WT cells was washed in minimal medium with glucose prior to inoculation in 5 mL of the same medium. Cultures were grown for 48 h unless otherwise noted, such as in Fig. 1. 1 mL of culture was centrifuged at 20,000 × *g* for 2 min, and the supernatant was frozen for later analysis. Prior to analysis, the supernatant was again centrifuged, followed by passing through a 0.22 μm PTFE filter. The samples were analyzed by HPLC using a Rezex ROA-Organic Acid H+ column with a flow rate of 0.8 mL per minute at 80°C, with 5 mM sulfuric acid as the mobile phase. OAs were detected by UV at 210 nm or refractive index and quantified using standard curves made from purchased individual OAs. Glucose remaining in each sample was approximated by dividing the glucose peak area of the sample by the glucose peak area of uninoculated minimal medium with glucose.

### Determination of strain growth rates.

Bacterial strains were streaked on TYG agar and grown at 37°C for 2–3 days. A single isolated colony of each strain was picked into TYG liquid medium and grown overnight. An amount comparable to 10 μL of WT overnight culture was washed in either 500 μL TYG or minimal medium with glucose before resuspension in 1 mL of the same medium. 200 μL of each strain/medium were added in triplicate to a 96-well plate. Cells were grown anaerobically in a Sunrise microplate reader (Tecan, Switzerland) with temperature set at 36–37°C. OD_600_ was read every 5 min for 2 days.

### Quantification of gene expression.

Bacterial strains were streaked on TYG agar and grown anaerobically at 37°C for 2 days. Three isolated colonies from each strain were picked into TYG liquid medium and grown overnight. OD_600_ was measured and cell amounts comparable to 50 μL of the WT culture were used for the assay. After removing the spent medium, cells were washed in 500 μL minimal medium with glucose before resuspension in 5 mL of the same medium. OD_600_ was periodically measured, and 1.5 mL sample was taken at OD_600_ 0.6–0.8. This was centrifuged for 3 min at 12,400 × *g* before removing supernatant and resuspending in 750 μL RNA-Later (Ambion). Samples were then kept at −20°C until extraction of RNA with the RNeasy minikit (Qiagen). 1 μg of RNA was treated with DNase I (Thermo Scientific) and then first strand cDNA was immediately synthesized with random hexamer primers using the RevertAid kit (Thermo Scientific) as directed.

qRT-PCR analysis of BT3692-3 gene expression was performed using a Mx3005P thermal cycler (Agilent) and DyNAmo ColorFlash SYBR green qPCR Kit (Thermo Scientific). Primers are listed in Table S8. Samples were run in triplicate with 0.5 μM primers and 10 ng cDNA, with the following cycling conditions: 95° C for 7 min, followed by 40 cycles of 95° C for 10 s and 60° C for 30 s. This protocol was followed by a melting curve to analyze amplicon purity. Quantification cycle (C_q_) values for BT3692 and BT3693 primer sets were normalized to the reference gene BT0899 (*gyrA*) and to the wild-type sample using the ΔΔCq method.
