# Supplementary material for: Investigation and Alteration of Organic Acid Synthesis Pathways in the Mammalian Gut Symbiont Bacteroides thetaiotaomicron
Source: Microbiol Spectr. 2022 Feb 23;10(1):e02312-21. doi: 10.1128/spectrum.02312-21 (PMC8865466; doi:10.1128/spectrum.02312-21)
Supplement: SUPPLEMENTAL FILE 1 — Supplemental material. Download SPECTRUM02312-21_Supp_1_seq4.pdf, PDF file, 0.9 MB [file spectrum02312-21_supp_1_seq4.pdf]

Supplemental Information: Investigation and alteration of organic acid synthesis pathways in the mammalian gut symbiont *Bacteroides thetaiotaomicron*

Nathan T. Porter, and Johan Larsbrink

2 Supplemental Figures

8 Supplemental Tables

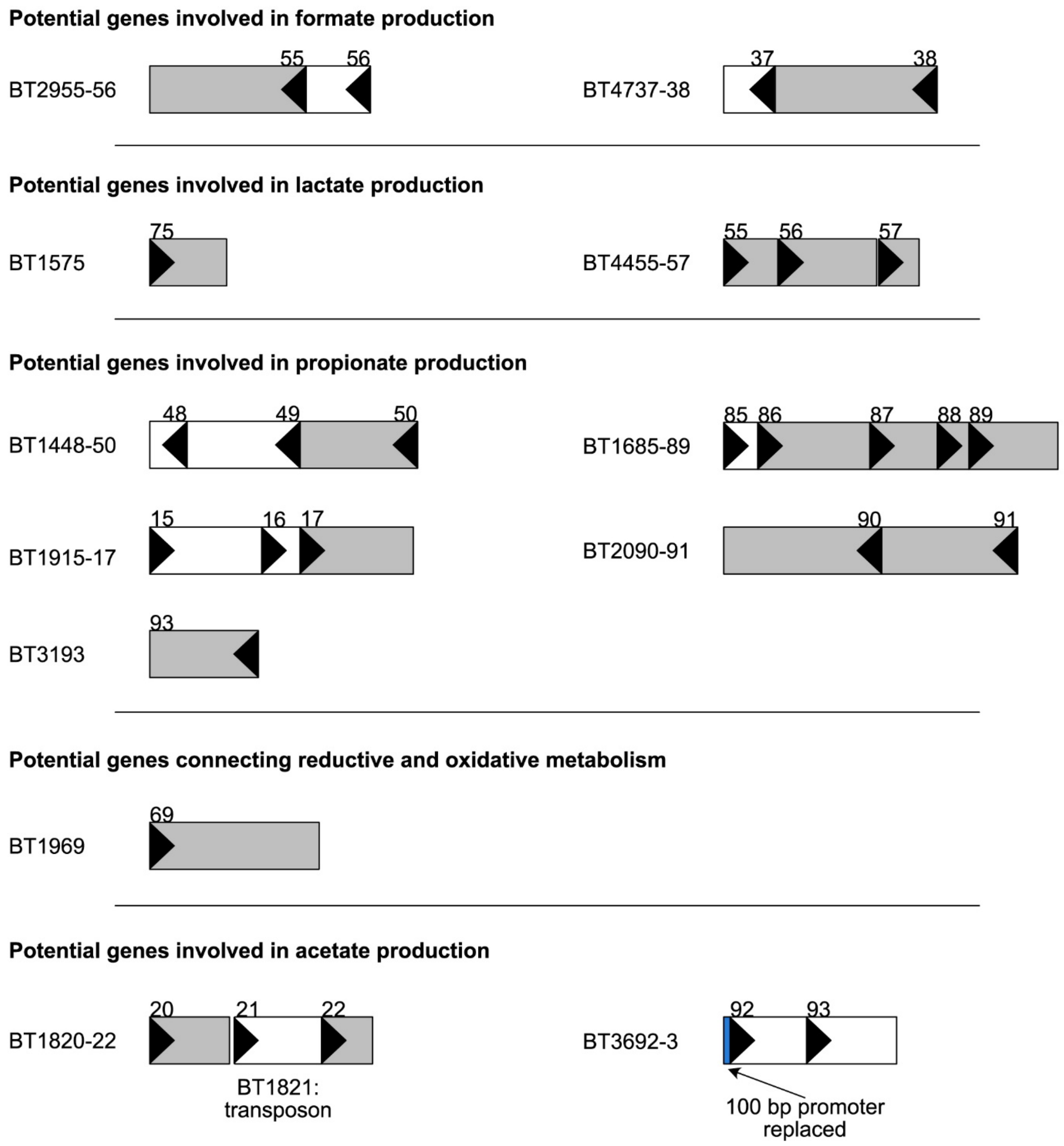

Figure S1. Maps of genetic loci investigated in this study, drawn to scale. Loci are organized by metabolic pathway in which they are putatively involved, with corresponding locus tags shown. Gray boxes indicate genes which were deleted in this study. The blue box indicates the BT3692-93 promoter replaced in this study.

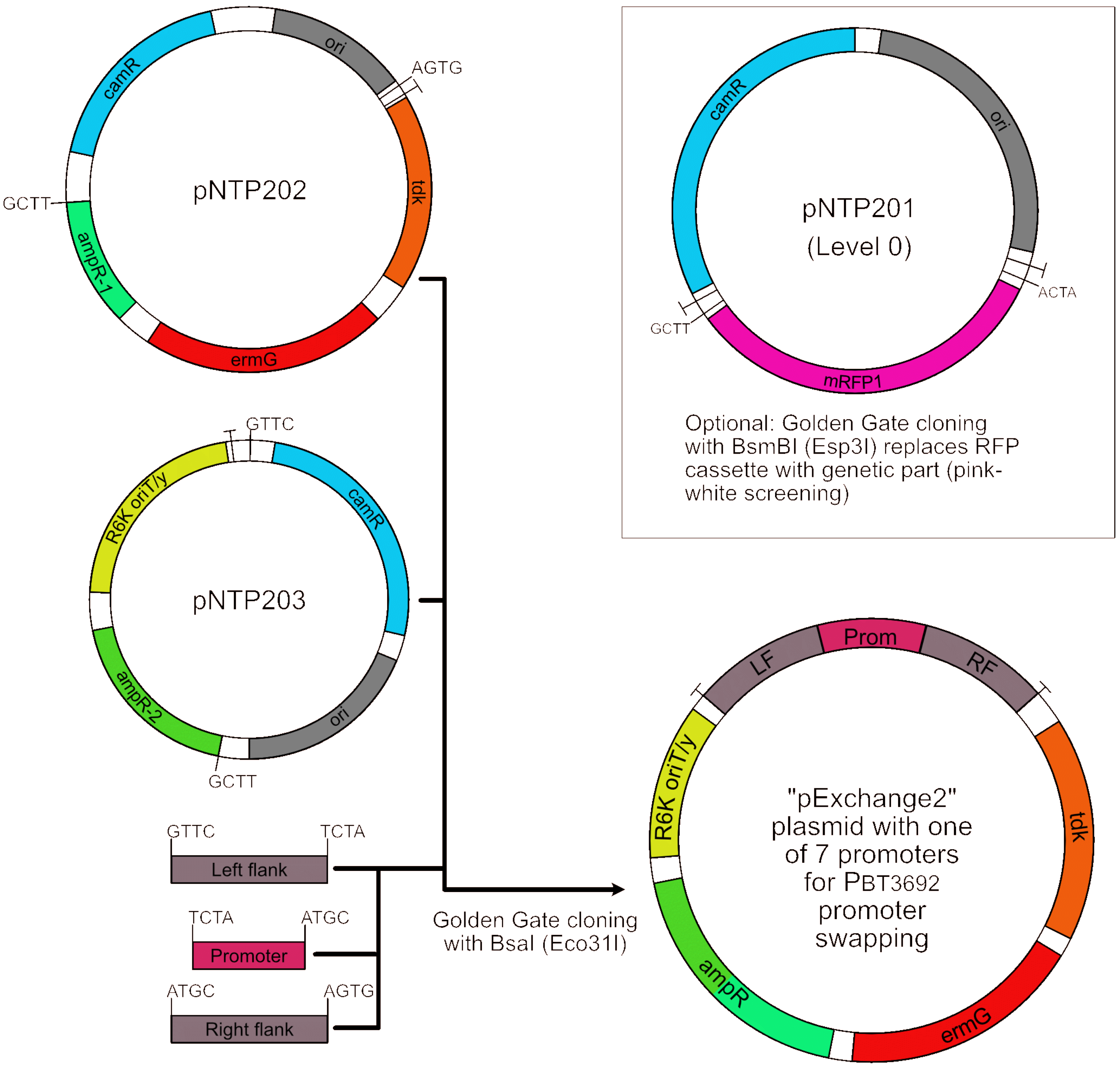

Figure S2. New Golden Gate cloning plasmid set for *Bacteroides* non-replicating plasmids based on pExchange-tdk. Optionally, genetic parts can first be incorporated into pNTP201 with Golden Gate cloning and BsmBI. Then the final "pExchange2" vector is assembled from pNTP202, pNTP203, and inserts (PCR amplicons, oligonucleotides, or plasmids) with BsaI-based Golden Gate cloning. 4-nucleotide overhang sequences are shown for each part used here. Flanking termination sequences are also shown with a "T" on pNTP201-3 and the final plasmid.

Table S1. **Organic acid concentrations (mM ± standard deviation) and glucose remaining over time for wild-type *B. thetaiotaomicron* (n = 3)**

Time (hr)	Acetate	α-keto glutarate	Formate	Lactate	Malate	Prop-ionate	Pyruvate	Succ-inate	Glucose (%)[a]
0	ND[b]	ND	ND	ND	ND	ND	ND	ND	96.4 ± 4.4
6	1.81 ± 0.32	0.02 ± 0.00	1.18 ± 0.18	ND	0.68 ± 0.01	1.01 ± 0.07	ND	0.90 ± 0.16	90.5 ± 3.2
7	2.69 ± 0.48	0.03 ± 0.00	1.84 ± 0.19	ND	0.73 ± 0.03	1.09 ± 0.05	ND	1.51 ± 0.26	81.1 ± 4.1
8	4.26 ± 0.66	0.03 ± 0.00	2.46 ± 0.38	ND	0.83 ± 0.06	1.23 ± 0.07	ND	2.33 ± 0.40	68.9 ± 4.7
12	9.77 ± 0.50	ND	6.48 ± 0.17	2.69 ± 0.56	1.90 ± 0.12	2.70 ± 0.10	0.20 ± 0.00	5.29 ± 0.30	25.2 ± 4.5
24	14.07 ± 0.43	0.05 ± 0.00	7.73 ± 0.07	7.87 ± 0.17	2.39 ± 0.02	5.07 ± 0.07	0.11 ± 0.01	6.93 ± 0.07	ND
30	14.20 ± 0.42	0.04 ± 0.00	7.64 ± 0.09	7.76 ± 0.10	2.23 ± 0.02	5.34 ± 0.03	0.08 ± 0.00	7.00 ± 0.08	ND
36	14.27 ± 0.12	0.04 ± 0.00	7.61 ± 0.03	7.59 ± 0.10	2.12 ± 0.01	5.82 ± 0.05	0.07 ± 0.00	7.22 ± 0.13	ND
48	14.85 ± 0.62	0.03 ± 0.03	7.17 ± 0.44	6.79 ± 0.51	2.03 ± 0.20	6.21 ± 0.16	0.23 ± 0.30	7.56 ± 0.46	ND
72	15.25 ± 0.54	0.04 ± 0.00	7.75 ± 0.18	7.34 ± 0.14	1.57 ± 0.02	6.16 ± 0.25	0.02 ± 0.00	7.99 ± 0.20	ND

[a] Glucose calculated as percent remaining glucose relative to minimal medium with glucose

[b] ND: Not detected; butyrate and fumarate were not detected in any samples

Table S2. Genes putatively involved in the metabolic pathways outlined in Figure 2.

Gene ID	Derived predicted annotation	GenBank Accession	Uniprot Entry	Size (aa)	TH[a]	Locus	TSS[b]	Putative source node	Putative destination node	RPKM EEP[c]	RPKM MEP[d]	RPKM Stat[e]	Reference
BT0309	Dihydrolipoamide dehydrogenase (E3 component, 2-oxoglutarate dehydrogenase complex)	NP_809222	Q8AB03	447		BT0309-BT0313	Yes	2-oxo glutarate	Succinyl-CoA	1.92	3.92	59.63	
BT0310	Lipoate-protein ligase A	NP_809223	Q8AB02	239		BT0309-BT0313		2-oxo glutarate	Succinyl-CoA	1.25	2.69	23.65	
BT0311	Dihydrolipoamide succinyltransferase (E2 component, 2-oxoglutarate dehydrogenase complex)	NP_809224	Q8AB01	456		BT0309-BT0313	Yes	2-oxo glutarate	Succinyl-CoA	2.19	8.57	95.24	
BT0312	2-oxoglutarate decarboxylase (E1 component, 2-oxoglutarate dehydrogenase complex)	NP_809225	Q8AB00	678		BT0309-BT0313		2-oxo glutarate	Succinyl-CoA	2.74	6.21	81.91	
BT0313	Flavodoxin associated with 2-oxoglutarate dehydrogenase complex	NP_809226	Q8AAZ9	171		BT0309-BT0313				2.74	10.65	155.37	
BT0329	2-oxoglutarate/2-oxoacid ferredoxin oxidoreductase subunit gamma	NP_809242	Q8AAY3	180		BT0329-BT0333		2-oxo glutarate	Succinyl-CoA	53.59	68.99	37.83	
BT0330	2-oxoglutarate/2-oxoacid ferredoxin oxidoreductase subunit beta	NP_809243	Q8AAY2	235		BT0329-BT0334	Yes	2-oxo glutarate	Succinyl-CoA	40.46	46.53	26.05	
BT0331	2-oxoglutarate/2-oxoacid ferredoxin oxidoreductase subunit alpha	NP_809244	Q8AAY1	295		BT0329-BT0334		2-oxo glutarate	Succinyl-CoA	43.77	46.77	24.72	
BT0332	2-oxoglutarate/2-oxoacid ferredoxin oxidoreductase subunit alpha	NP_809245		59		BT0329-BT0334		2-oxo glutarate	Succinyl-CoA	51.56	57.49	29.21	
BT0333	2-oxoglutarate/2-oxoacid ferredoxin oxidoreductase subunit delta	NP_809246	Q8AAX9	75		BT0329-BT0334	BT0334	2-oxo glutarate	Succinyl-CoA	65.06	79.51	37.66	
BT0644	Pyruvate phosphate dikinase	NP_809557	Q8AA21	906			Yes	Pyruvate	PEP	11.98	7.82	15.96	
BT0787	Succinyl-CoA synthetase alpha chain	NP_809700	Q8A9M8	286		BT0787-BT0788		Succinate	Succinyl-CoA	0.20	0.59	4.07	
BT0788	Succinyl-CoA synthetase beta chain	NP_809701	Q8A9M7	376		BT0787-BT0788	Yes	Succinate	Succinyl-CoA	0.49	0.62	5.11	
BT1196	Pyruvate carboxylase	NP_810109	Q8A8H1	591			Yes	Pyruvate	Oxalo acetate	1.28	3.59	46.60	
BT1448	Biotin-dependent carboxylase, biotin carboxyl carrier protein	NP_810361	Q8A7S6	174		BT1448-BT1450				0.57	0.83	13.84	

BT1449	Biotin-dependent carboxylase, biotin carboxylase subunit	NP_810362	Q8A7S5	503		BT1448-BT1450				0.59	0.63	13.67	
BT1450	Biotin-dependent carboxylase, carboxytransferase subunit	NP_810363	Q8A7S4	514		BT1448-BT1450	Yes			0.52	0.59	13.28	This work
BT1575	D-lactate dehydrogenase	NP_810488	Q8A7E9	333				Pyruvate	Lactate	2.87	4.99	14.15	This work
BT1685	Methylmalonyl-CoA epimerase	NP_810598	Q8A740	134		BT1685-BT1689	Yes	Methyl malonyl-CoA	Methylmalonyl-CoA	89.81	72.00	27.45	
BT1686	Methylmalonyl-CoA decarboxylase (alpha subunit)	NP_810599	Q8A739	517		BT1685-BT1689		Methyl malonyl-CoA	Propionyl-CoA	96.91	65.63	23.72	This work, (1)
BT1687	Methylmalonyl-CoA decarboxylase (membrane associated subunit)	NP_810600	Q8A738	306	1	BT1685-BT1689		Methyl malonyl-CoA	Propionyl-CoA	87.26	53.66	14.83	This work, (1)
BT1688	Methylmalonyl-CoA decarboxylase (biotin carboxylase component)	NP_810601	Q8A737	144		BT1685-BT1689		Methyl malonyl-CoA	Propionyl-CoA	98.86	65.01	18.77	This work, (1)
BT1689	Methylmalonyl-CoA decarboxylase (beta subunit)	NP_810602	Q8A736	386	10	BT1685-BT1689		Methyl malonyl-CoA	Propionyl-CoA	77.89	62.45	18.84	This work, (1)
BT1696	Oxaloacetate decarboxylase, Na+ transporting (beta subunit)	NP_810609	Q8A729	411	9	BT1696-BT1698		Oxalo acetate	Pyruvate	8.78	7.22	5.11	
BT1697	Oxaloacetate decarboxylase, Na+ transporting (alpha subunit)	NP_810610	Q8A728	609		BT1696-BT1698		Oxalo acetate	Pyruvate	7.89	8.79	6.35	
BT1698	Oxaloacetate decarboxylase, Na+ transporting (gamma subunit)	NP_810611	Q8A727	86	2	BT1696-BT1698	Yes	Oxalo acetate	Pyruvate	13.32	11.87	14.88	
BT1747	Pyruvate ferredoxin oxidoreductase (PFOR)	NP_810660	Q8A6X8	1185				Pyruvate	Acetyl-CoA	74.55	99.46	49.95	(2)
BT1820	Pyruvate oxidase (split by transposable element BT1821)	NP_810733	Q8A6Q6	362		BT1820-BT1822	Yes	Pyruvate	Acetate	0.89	4.10	47.66	This work
BT1822	Pyruvate oxidase (split by transposable element BT1821)	NP_810735	Q8A6Q5	228		BT1820-BT1822		Pyruvate	Acetate	0.24	1.01	10.60	This work
BT1915	Biotin-dependent carboxylase, biotin carboxylase subunit	NP_810828	Q8A6G9	503		BT1915-BT1917	Yes	Pyruvate	Oxalo acetate	73.37	57.03	40.94	
BT1916	Biotin-dependent carboxylase, biotin carboxyl carrier protein	NP_810829	Q8A6G8	169		BT1915-BT1917				81.37	63.85	51.74	
BT1917	Biotin-dependent carboxylase, carboxytransferase subunit	NP_810830	Q8A6G7	511		BT1915-BT1917		Succinate	Propionate	94.73	73.61	62.12	This work
BT1969	Malate dehydrogenase (NADP-dependent)	NP_810882	Q8A6B5	763			Yes	Malate	Pyruvate	14.54	19.15	19.05	This work

BT1972	Phosphoenolpyruvate synthase	NP_810885	Q8A6B3	990			Yes	Pyruvate	PEP	5.27	4.02	3.25	
BT2070	Citrate synthase	NP_810983	Q8A616	447		BT2070-BT2072		Acetyl-CoA	Citrate	8.70	15.50	70.24	
BT2071	Isocitrate dehydrogenase	NP_810984	Q8A615	396		BT2070-BT2072		Isocitrate	2-oxo glutarate	5.89	12.45	63.38	
BT2072	Aconitase	NP_810985	Q8A614	747		BT2070-BT2072		Citrate	Isocitrate	5.54	11.85	54.16	
BT2090	Methylmalonyl-CoA mutase (large subunit)	NP_811003	Q8A5Z6	715		BT2090-BT2091		Succinate	Propionate	17.89	24.16	27.71	This work
BT2091	Methylmalonyl-CoA mutase (small subunit)	NP_811004	Q8A5Z5	633		BT2090-BT2091	Yes	Succinate	Propionate	17.75	22.69	21.46	This work
BT2256	Fumarase, class I, homodimeric	NP_811169	Q8A5I3	544			Yes	Malate	Fumarate	94.12	140.72	79.26	(3)
BT2510	Malate dehydrogenase (NAD-dependent)	NP_811423	Q8A4T7	333			Yes	Oxalo acetate	Malate	24.30	56.09	67.76	
BT2790	Phosphoenolpyruvate carboxykinase	NP_811702	Q8A414	535			Yes	PEP	Oxalo acetate	523.59	766.60	316.40	
BT2836	2-oxoglutarate/2-oxoacid ferredoxin oxidoreductase subunit alpha	NP_811748	Q8A3W8	616		BT2836-BT2837	Yes	2-oxo glutarate	Succinyl-CoA	24.61	24.81	9.56	
BT2837	2-oxoglutarate/2-oxoacid ferredoxin oxidoreductase subunit beta	NP_811749	Q8A3W7	336		BT2836-BT2837		2-oxo glutarate	Succinyl-CoA	23.39	22.28	9.49	
BT2841	Pyruvate kinase	NP_811753	Q8A3W3	485				PEP	Pyruvate	70.72	47.98	19.38	
BT2955	Formate acetyltransferase 2 [Pyruvate formate-lyase]	NP_811867	Q8A3J9	714		BT2955-BT2956		Pyruvate	Formate	0.04	0.07	0.09	This work, (2)
BT2956	Pyruvate formate lyase activating enzyme	NP_811868	Q8A3J8	299		BT2955-BT2956		Pyruvate	Formate	0.05	0.00	0.09	
BT3053	Fumarate reductase (cytochrome b subunit)	NP_811965	Q8A3A1	234	5	BT3053-BT3055	Yes	Fumarate	Succinate	165.87	220.34	63.42	(4)
BT3054	Fumarate reductase (flavoprotein subunit)	NP_811966	Q8A3A0	659		BT3053-BT3055		Fumarate	Succinate	142.68	198.90	58.45	(4)
BT3055	Fumarate reductase (iron sulfur protein subunit)	NP_811967	Q8A399	251		BT3053-BT3055		Fumarate	Succinate	116.09	157.01	49.08	(4)
BT3186	Dihydrolipoamide dehydrogenase (E3 subunit, 2-oxoacid dehydrogenase complex)	NP_812098	Q8A2W9	447			BT3185			5.04	4.76	2.05	
BT3193	Coenzyme A transferase	NP_812105	Q8A2W2	499				Propionyl-CoA	Succinyl-CoA	28.38	21.45	5.09	This work
BT3692	Phosphate acetyltransferase	NP_812603	Q8A1G9	339		BT3692-BT3693	Yes	Acetyl-CoA	Acetyl phosphate	33.06	38.00	7.96	This work
BT3693	Acetate kinase	NP_812604	Q8A1G8	399		BT3692-BT3693		Acetyl phosphate	Acetate	28.12	32.05	8.46	This work
BT3755	Acetyl-coenzyme A synthetase	NP_812666	Q8A1B1	551			BT3757	Acetate	Acetyl-CoA	17.51	11.31	13.59	
BT3911	Malate dehydrogenase (NAD-dependent)	NP_812822	Q8A0W0	313			Yes	Oxalo acetate	Malate	200.37	543.45	473.80	

BT4455	L-lactate dehydrogenase, Fe-S oxidoreductase subunit	NP_813366	Q89ZC2	246		BT4455-BT4457	Yes	Lactate	Pyruvate	22.41	31.12	63.69	This work
BT4456	L-lactate dehydrogenase, Iron-sulfur cluster-binding subunit	NP_813367	Q89ZC1	461		BT4455-BT4457		Lactate	Pyruvate	20.53	25.60	61.81	This work
BT4457	L-lactate dehydrogenase, hypothetical protein subunit	NP_813368	Q89ZC0	193		BT4455-BT4457		Lactate	Pyruvate	15.77	21.79	60.98	This work
BT4737	Pyruvate formate lyase activating enzyme	NP_813648	Q89YJ4	242		BT4737-BT4738		Pyruvate	Formate	39.20	33.02	3.22	
BT4738	Formate acetyltransferase [Pyruvate formate-lyase]	NP_813649	Q89YJ3	742		BT4737-BT4738	Yes	Pyruvate	Formate	186.40	117.01	16.28	This work, (2)

[a] TH: Predicted transmembrane helices (IMG)

[b] TSS: 5' transcriptional start site (from Theta-Base, (5))

[c] RPKM EEP: Normalized gene expression of *Bt* in early exponential phase (Theta-Base, (5))

[d] RPKM MEP: Normalized gene expression of *Bt* in mid exponential phase (Theta-Base, (5))

[e] RPKM Stat: Normalized gene expression of *Bt* in stationary phase (Theta-Base, (5))

Table S3. Growth summary data for strains grown in rich medium (TYG) and minimal medium (MMG)

Strain	TYG Max OD600[a]	TYG Specific Rate[b]	MMG Max OD600	MMG Specific Rate
**Growth curve summary data from Figures 3-7; n = 3 except WT, n = 6**				
WT	1.36 ± 0.06	0.1647 ± 0.0110	1.09 ± 0.11	0.0813 ± 0.0253
ΔBT2955	1.32 ± 0.09	0.1712 ± 0.0065	1.00 ± 0.03	0.0803 ± 0.0290
ΔBT4738	1.26 ± 0.09	0.1619 ± 0.0075	0.98 ± 0.07	0.0668 ± 0.0243
ΔBT2955/4738	1.28 ± 0.09	0.1608 ± 0.0120	0.99 ± 0.08	0.0669 ± 0.0248
ΔBT1575	1.42 ± 0.04	0.1644 ± 0.0213	1.15 ± 0.11	0.0812 ± 0.0348
ΔBT4455	1.42 ± 0.03	0.1593 ± 0.0181	1.15 ± 0.11	0.0802 ± 0.0319
ΔBT1575/4455-7	1.43 ± 0.02	0.1632 ± 0.0155	1.17 ± 0.11	0.0861 ± 0.0357
ΔBT1450	1.34 ± 0.08	0.1672 ± 0.0091	0.99 ± 0.05	0.0758 ± 0.0232
ΔBT1686-9	1.30 ± 0.04	0.1286 ± 0.0093	0.96 ± 0.05	0.0797 ± 0.0184
ΔBT1917	1.35 ± 0.05	0.1816 ± 0.0166	1.02 ± 0.03	0.0814 ± 0.0226
ΔBT2090-1	1.30 ± 0.06	0.1566 ± 0.0217	1.04 ± 0.13	0.0861 ± 0.0410
ΔBT3193	1.26 ± 0.07	0.1410 ± 0.0116	0.33 ± 0.19	0.0059 ± 0.0040
ΔBT1969	1.43 ± 0.02	0.1534 ± 0.0075	1.12 ± 0.14	0.0750 ± 0.0309
ΔBT1820	1.42 ± 0.02	0.1538 ± 0.0063	1.15 ± 0.11	0.0814 ± 0.0285
ΔBT1822	1.39 ± 0.05	0.1502 ± 0.0200	1.20 ± 0.09	0.0846 ± 0.0275
ΔBT1820/1822	1.41 ± 0.02	0.1459 ± 0.0168	1.15 ± 0.07	0.0793 ± 0.0273
**Growth curve summary data from Figure 8; n = 3**				
WT	1.45 ± 0.02	0.1463 ± 0.0044	0.90 ± 0.05	0.0406 ± 0.0049
P_BfP2E3	1.29 ± 0.02	0.0874 ± 0.0085	0.90 ± 0.08	0.0296 ± 0.0066
P_BfP5E4	1.40 ± 0.02	0.1354 ± 0.0069	0.92 ± 0.03	0.0402 ± 0.0029

[a] OD600 = optical density at 600 nm

[b] Specific rate: (Maximum - Minimum OD600) / time

Table S4. Organic acid concentrations (mM ± standard deviation) at 48 hours for the wild-type (WT), various mutant strains and their complements (n = 3)

Strain	Acetate	Formate	Lactate	Malate	Propionate	Succinate
WT	16.48 ± 0.35	7.55 ± 0.28	2.09 ± 0.25	ND[a]	7.74 ± 0.64	9.47 ± 0.64
ΔBT1686-9	17.60 ± 0.28	8.75 ± 1.26	2.26 ± 0.32	1.52 ± 0.08	ND	17.33 ± 0.42
Comp[b] 1685-9	16.67 ± 0.19	8.08 ± 0.43	2.51 ± 0.63	ND	8.70 ± 0.26	8.95 ± 0.77
ΔBT2090-1	16.82 ± 0.27	10.13 ± 0.85	2.62 ± 0.63	1.56 ± 0.06	ND	17.49 ± 0.32
Comp 2090-1	17.09 ± 0.07	7.25 ± 1.48	1.47 ± 0.21	ND	8.52 ± 1.58	8.61 ± 1.90
ΔBT4738	15.04 ± 0.07	1.93 ± 0.66	1.16 ± 0.06	ND	7.77 ± 0.92	12.50 ± 0.16
Comp 4738	16.00 ± 0.51	6.45 ± 1.25	1.73 ± 0.37	0.55 ± 0.95	9.27 ± 1.26	9.93 ± 1.66

[a] Not detected. α-ketoglutarate or pyruvate were also not detected in any strain or sample.

[b] Comp: Mutant strain from the row above complemented with the listed genes

Table S5. Organic acid concentrations (mM ± standard deviation) at 48 hours for the wild-type (WT) and ΔBT1969 strains (n = 6)

Strain	Acetate	Formate	Lactate	Malate	Propionate	Succinate
WT	14.62 ± 0.42	7.72 ± 0.75	1.83 ± 0.13	ND[a]	8.76 ± 0.60	6.11 ± 0.48
ΔBT1969	13.03 ± 0.25	9.22 ± 1.14	1.40 ± 0.11	1.39 ± 0.68	8.30 ± 0.57	8.43 ± 0.56

[a] Not detected. α-ketoglutarate or pyruvate were also not detected in any strain or sample.

Table S6. Organic acid concentrations (mM ± standard deviation) at 48 hours for the wild-type (WT) and the promoter replacement strains (n = 3)

Strain	Acetate	Formate	Lactate	Malate	Propionate	Succinate
WT	17.43 ± 0.17	11.60 ± 0.52	2.26 ± 0.05	1.60 ± 0.01	10.92 ± 0.37	6.84 ± 0.22
P_BfP2E3	17.13 ± 0.20	16.76 ± 14.10	3.13 ± 1.22	0.64 ± 1.11	9.46 ± 0.26	6.79 ± 1.01
P_BfP5E4	17.12 ± 0.25	11.26 ± 0.29	2.44 ± 0.17	1.54 ± 0.02	9.89 ± 0.10	7.23 ± 0.16

Not detected for any strain or sample: α-ketoglutarate or pyruvate

Table S7. Strains and plasmids used in this study.

Name	Genotype	Ref.	Notes
**Strains**			
WT *Bt*	*tdk*- (ΔBT2275)	(6)	Parent strain of other *Bt* strains; derived from ATCC 29148/VPI-5482
ΔBT2955	*tdk*- ΔBT2955		
ΔBT4738	*tdk*- ΔBT4738		
ΔBT2955/4738	*tdk*- ΔBT2955/4738		
ΔBT1575	*tdk*- ΔBT1575		
ΔBT4455-7	*tdk*- ΔBT4455-7		
ΔBT1575/4455-7	*tdk*- ΔBT1575/4455-7		
ΔBT1450	*tdk*- ΔBT1450		
ΔBT1686-9	*tdk*- ΔBT1686-9	(1)	
ΔBT1917	*tdk*- ΔBT1917		
ΔBT2090-1	*tdk*- ΔBT2090-1		
ΔBT3193	*tdk*- ΔBT3193		
ΔBT1969	*tdk*- ΔBT1969		
ΔBT1820	*tdk*- ΔBT1820		
ΔBT1822	*tdk*- ΔBT1822		
ΔBT1820/1822	*tdk*- ΔBT1820/1822		
Comp 1685-9	*tdk*- ΔBT1686-9 ::pNBU2-bla-tetQb::BT1685-9		pNBU2-bla-tetQb chromosomally integrates at one of two tRNAser sites in *Bt*
Comp 2090-1	*tdk*- ΔBT2090-1 ::pNBU2-bla-tetQb::BT2090-1		pNBU2-bla-tetQb chromosomally integrates at one of two tRNAser sites in *Bt*
Comp 4738	*tdk*- ΔBT4738 ::pNBU2-bla-tetQb::BT4738		pNBU2-bla-tetQb chromosomally integrates at one of two tRNAser sites in *Bt*
PBT3692::P_BfP2E3	*tdk*- PBT3692::P_BfP2E3		Promoter replaces 100 bp sequence immediately upstream of BT3692
PBT3692::P_BfP5E4	*tdk*- PBT3692::P_BfP5E4		Promoter replaces 100 bp sequence immediately upstream of BT3692
Escherichia coli S17-1 λ*pir*			Shuttles plasmids to *Bacteroides*
Escherichia coli BW27343	*pir*+ derivative of DH5α		Transformation and maintenance of plasmids; CGSC strain #7924
**Plasmids**			
pExchange-*tdk*		(6)	Counterselectable suicide plasmid for gene deletions
pExchange::ΔBT2955			Deletion of BT2955
pExchange::ΔBT4738			Deletion of BT4738
pExchange::ΔBT1575			Deletion of BT1575
pExchange::ΔBT4455-7			Deletion of BT4455-7
pExchange::ΔBT1450			Deletion of BT1450
pExchange::ΔBT1686-9		(1)	Deletion of BT1686-9
pExchange::ΔBT1917			Deletion of BT1917

pExchange::ΔBT2090-1			Deletion of BT2090-1
pExchange::ΔBT3193			Deletion of BT3193
pExchange::ΔBT1969			Deletion of BT1969
pExchange::ΔBT1820			Deletion of BT1820
pExchange::ΔBT1822			Deletion of BT1822
pNBU2-bla-tetQb		(7)	pNBU2-bla-tetQb chromosomally integrates at one of two tRNAser sites in *Bt*
pNBU2-bla-tetQb::BT1685-9			BT1685-9 gene complementation
pNBU2-bla-tetQb::BT2090-1			BT2090-1 gene complementation
pNBU2-bla-tetQb::BT4738			BT4738 gene complementation
pNTP201			Vector for integrating Golden Gate-compatible parts
pNTP202			pExchange2, part A
pNTP203			pExchange2, part B
pNTP221			pExchange2 with PBT3692::PBT1431 for PBT3692 replacement
pNTP224			pExchange2 with PBT3692::BfP3E1/RBS8 for PBT3692 replacement
pNTP225			pExchange2 with PBT3692::BfP2E2/RBS8 for PBT3692 replacement
pNTP226			pExchange2 with PBT3692::P_BfP2E3/RBS8 for PBT3692 replacement
pNTP227			pExchange2 with PBT3692::P_BfP5E4/RBS8 for PBT3692 replacement
pNTP228			pExchange2 with PBT3692::PBT1925 for PBT3692 replacement
pNTP230			pExchange2 with PBT3692::PBT3110 for PBT3692 replacement
pNTP231			pExchange2 with PBT3692::PBT3321 for PBT3692 replacement
pNTP232			pExchange2 with PBT3692::PBT4618 for PBT3692 replacement

Ref.: Reference

Table S8. Primers, promoters and ribosome binding site used in this study.

Name	Notes	Sequence (restriction sites underlined)
**Deletion constructs**		
BT1450 left F		GCGTCTAGAGCCCAGATGAACCACATTG
BT1450 left R		TAATAAATGGAAAATGGAAAGTTG
BT1450 right F		TTTCCATTTTCCATTTATTACATAGGCTGTTTGTATTAATGTTATG
BT1450 right R		GCGGTCGACCATATTTCAGACGGGCGTAAC
BT1917 left F		GCGTCTAGAGCTTTATCACCAAGAACGG
BT1917 left R		CATGATGCAATCCGTTTTTAGTA
BT1917 right F		TAAAAACGGATTGCATCATGTAAGTTATAGGCGATTACCTATAA
BT1917 right R		GCGGTCGACCGGTCCAATAAGTAGGGTG
BT2090 left F		GCGTCTAGAGACAATCAATGCCAGCGTAG
BT2090 left R		TAAGTACGGAATTTAATCATTTTTTC
BT2091 right F		ATGATTAAATTCCGTACTTAGTCTGCCATAGTTTATTTAAAATTAAAAC
BT2091 right R		GCGGTCGACGGAGTGTTCGTCACAGC
BT3193 left F		GCGTCTAGACCTGTAACGAATCAATGACTTG
BT3193 left R		TAAGGCATGAAACGTCTGTC
BT3193 right F		GACAGACGTTTCATGCCTTACATAAATAGTATAATTTCGCGTTAAATG
BT3193 right R		GCGGTCGACCTTGTAGTTCAGGCATTATTCTG
BT4738 left F		GCGTCTAGACTTTGAATACCTCCGCAGC
BT4738 left R		TAATCTATTACTCTATATGATGATAAACG
BT4738 right F		TCATATAGAGTAATAGATTACATAAAATGATAAAATTAGAGGTTGTAG
BT4738 right R		GCGGTCGACGTACTCACGCTTCTTTTCCAG
BT1575 left F		GCGTCTAGACTTGCCCCTAACTGCAAAC
BT1575 left R		CATAATATTTTCTGTTTTTATTCTTATTAATAAACG
BT1575 right F		TAAAAACAGAAAATATTATGTAGAACCTTTACCTGACTTCCC
BT1575 right R		GCGGTCGACCAATAAGATATGTGAGTCCTTCAATG
BT1820 left F		GCGTCTAGAGTCTGCTGATGCTTGGAATAG
BT1820 left R		CATATTTCTAATAGGTTTAGGATTGATTCTTAAT
BT1820 right F		CTAAACCTATTAGAAATATGTAAAAGAGTTCTTTGAAATATTTGAAATTTAGTT
BT1820 right R		GCGGTCGACCTCTGACAACAAAGTAGGCTTC
BT1822 left F		GCGTCTAGAGCATACATTATATGATGTGGAAACAC
BT1822 left R		CATTAGTGTCCCATTAAAATTGGGA

BT1822 right outer F	Outer PCR used as template for BT1822 right flank	GCAATCCGGAAGAAGTACTGA
BT1822 right outer R	Outer PCR used as template for BT1822 right flank	GGAATATGTTGACGTCCCTAAGG
BT1822 right F		ATTTTAATGGGACACTAATGTGACAGTTCCTTCTTCAACGA
BT1822 right R		GCGGTCGACGTTTTGTCGGCTGAGGGT
BT2955 left F		GCGTCTAGACCCAGATAATCAATTCCTTTTCCG
BT2955 left R		TAAATCATATTCACTCTAAAAATCTTATATCATGA
BT2955 right F		TTTAGAGTGAATATGATTTACTCGTAATTCTTCATTTTTTGTTATTGTT
BT2955 right R		GCGGTCGACCCATTGTTTCTCTTGTATAACGGT
BT1969 left F		GCGTCTAGACCATGCTATGTACTTCCCGATA
BT1969 left R		GGTTATTTTAGCCATGGTATTGGTA
BT1969 right F		ATACCATGGCTAAAATAACCTGAGAAGGCCGTTATCAAAATC
BT1969 right R		GCGGTCGACCGTTTGACTTACCACGTGG
BT4455-57 left F		GCGTCTAGACCGCAATGCTATAAAGTAGAAGTG
BT4455-57 left R		CATAATTATATTACGTAAAATTTAGATGCATCCT
BT4455-57 right F		TTTTACGTAATATAATTATGGTCATATTGACGTAACTCCGC
BT4455-57 right R		GCGGTCGACGACCTTTATCGGACAGGAGAC
**Complementations**		
BT1685-9 F		GCGTCTAGAGCTTTATTCCCCAATAATTAAATGCA
BT1685-9 R		GCGGTCGACGAGAAGATAACATAATGATAAGTGCG
BT2090-1 F		GCGTCTAGAGCTTTAAGTTGCATGGAGATTCT
BT2090-1 R		GCGGTCGACCATCCCGCCTTCAGCC
BT4738 F		GCGTCTAGACTAATCTTTTCCTAAGGATAGCAATT
BT4738 R		GCGGTCGACATAGAGTAATAGATTACATACGTTCGTG
**Golden Gate flank primers**		
PBT3692 GG left-F		GCGGGTCTCCGTTCTGTAGCTTGTCGGACAAGTG
PBT3692 GG left-R		GCGGGTCTCGTAGATATTCTCCAAGTCTAAAATATTATAATATTTAACACT
PBT3692 GG right-F		GCGGGTCTCCATGCTTAATTTAATCAACCAAATCGTG
PBT3692 GG right-R		GCGGGTCTCGCACTTTCTTACCATACTCGGGAGC
**qPCR primers**		
BT0899 qPCR-F	*gyrA* as reference gene	CCATGCGTTATACGGAGGCT
BT0899 qPCR-R	*gyrA* as reference gene	TCACCTTCGGCTCTGTCAAC
BT3692 qPCR-F		CCGCAACACAACAGGCAA
BT3692 qPCR-R		ACACAAGTGATGCCCGGA
BT3693 qPCR-F		ATGCCGGTGCAGTAACATTC

BT3693 qPCR-R		TGATACGCCGGAAATACCCA
**Promoters and Ribosome Binding Sites (RBS)**		
PBT3692	Native promoter sequence; replaced by other promoters in this study	AAATTAGCCGGAATTGTAATGTGGATATTGCAAAATTGAGTACTTTTGCAGCGCAAATTACAAATCGTAACAAGGTTTATAATATTATTCAAATATATCT
P_BfP3E1	Promoter from pWW3852, (8)	GATAAAACGAAAGGCTCAGTCGAAAGACTGGGCCTTTCGTTTTACAATTGGGCTACCTTTTTTTTGTAAAAAAAAACCCCGCCCCTGACAGGGCGGGGTTTTTTTTTCACTTGAACTTTCAAATAATGTTCTTATAAAACCAGTGTCGAAAGAAACAAAGTAG
P_BfP2E2	Promoter from pWW3853, (8)	GATAAAACGAAAGGCTCAGTCGAAAGACTGGGCCTTTCGTTTTACAATTGGGCTACCTTTTTTTTGTAAAAAAAAACCCCGCCCCTGACAGGGCGGGGTTTTTTTTTCACTTGAACTTTCAAATAATGTTCTTATATATGCAGTGTCGAAAGAAACAAAGTAG
P_BfP2E3	Promoter from pWW3818, (8)	GATAAAACGAAAGGCTCAGTCGAAAGACTGGGCCTTTCGTTTTACAATTGGGCTACCTTTTTTTTGTTTTGTTTGCAATGGTTAATCTATTGTTAAAATTTAAAGTTTCACTTGAACTTTCAAATAATGTTCTTATATGTGCAGTGTCGAAAGAAACAAAGTAG
P_BfP5E4	Promoter from pWW3821, (8)	GATAAAACGAAAGGCTCAGTCGAAAGACTGGGCCTTTCGTTTTACAATTGGGCTACCTTTTTTTTGTTTTGTTTGCAATGGTTAATCTATTGTTAAAATTTAAAGTTTCACTTGAACTTTCAAATAATGTTCTTCTATTTGCAGTGTCGAAAGAAACAAAGTAG
RBS8	RBS used with Whitaker *et al.* 2017 synthetic promoters, (8)	GATCGTCCATCAATTTAAAATTTAAAAT
PBT1431	Promoter from *Bt*	GCCAATTCATGTATTTGCAAAAAATATCATGTACATTTGTGTTTCAAAAAACAGCAAAACGC
PBT1925	Promoter from *Bt*	GGGGTATTAGTATTGGGGAGCTTGCTCTCAAATAAAAGGTTACAGAATTGACGGTAGTGCGCCTCTGCCGGAATTTGAGGGTAAAATGGTTTAC
PBT3110	Promoter from *Bt*	GACCAAATTAAAAAATTGGAGGGTGACTGTTTTACAACCACCATCCTTTGCTGGTTATTGTTCTAAGTTAGTAATTTTATTCACTTTTACTTTTGCGTTTCAATTGTAAAAGCATCTTTTTTTCATAACTTTGTCCTCTGTATCCTTATTGTCTTTTTCTGTCGGAATATAACTTAATGATCCGGGATACTTCATAACAT
PBT3321	Promoter from *Bt*	AGTCGCAAAGATAAGATTTTTATTACATTTGCAGTGCACATTAACTTTCTGTTAAC
PBT4618	Promoter from *Bt*	TAGAGGTGTTCAGTAAAGAATGTGTTAGTCCAAACAGTCGTTTATTCCCCATTTATTATGTTGCCCAATAAATTTTCGTATTAATGAAACTTAAATCTGTCAGTGGTAAATGAGCGGTTGATTGGTGGCGGTCAAGCACCTATTTAGCACCTATAAGCATCCCTATACTTTTGCGGCATAAATTTTAATACGAAGAAAAA

References

1. Kovatcheva-Datchary P, Nilsson A, Akrami R, Lee YS, De Vadder F, Arora T, Hallen A, Martens E, Björck I, Bäckhed F. 2015. Dietary fiber-induced improvement in glucose metabolism is associated with increased abundance of *Prevotella*. Cell Metab 22:971–982.
2. Khademian M, Imlay JA. 2020. Do reactive oxygen species or does oxygen itself confer obligate anaerobiosis? The case of *Bacteroides thetaiotaomicron*. Mol Microbiol 1–15.
3. Lu Z, Sethu R, Imlay JA. 2018. Endogenous superoxide is a key effector of the oxygen sensitivity of a model obligate anaerobe. Proc Natl Acad Sci U S A 115:E3266–E3275.
4. Lu Z, Imlay JA. 2017. The fumarate reductase of *Bacteroides thetaiotaomicron*, unlike that of *Escherichia coli*, is configured so that it does not generate reactive oxygen species. MBio 8:1–11.
5. Ryan D, Jenniches L, Reichardt S, Barquist L, Westermann AJ. 2020. A high-resolution transcriptome map identifies small RNA regulation of metabolism in the gut microbe *Bacteroides thetaiotaomicron*. Nat Commun 11.
6. Bogachev A V., Bertsova Y V., Bloch DA, Verkhovsky MI. 2012. Urocanate reductase: Identification of a novel anaerobic respiratory pathway in *Shewanella oneidensis* MR-1. Mol Microbiol 86:1452–1463.
7. Martens EC, Chiang HC, Gordon JI. 2008. Mucosal glycan foraging enhances fitness and transmission of a saccharolytic human gut bacterial symbiont. Cell Host Microbe 4:447–57.
8. Whitaker WR, Shepherd ES, Sonnenburg JL. 2017. Tunable expression tools enable single-cell strain distinction in the gut microbiome. Cell 169:538-546.e12.
